# Impact of selected drugs and their binary mixtures on the germination of *Sorghum bicolor* (sorgo) seeds

**DOI:** 10.1007/s11356-018-2049-4

**Published:** 2018-04-29

**Authors:** Monika Wieczerzak, Błażej Kudłak, Jacek Namieśnik

**Affiliations:** Department of Analytical Chemistry, Faculty of Chemistry, Gdańsk University of Technology, 11/12 Narutowicza Str., 80-233 Gdańsk, Poland

**Keywords:** Phytotoxicity, Drug residues, Ecotoxicity, *Sorghum bicolor*, Plant bioassays, Model deviation ratio

## Abstract

The aim of this study was to assess the toxicological potential of binary drug mixtures and individual drugs under different pH conditions with different inorganic ion additions on the germination of *Sorghum bicolor* (sorgo) seeds. To assess whether the given drug mixtures were more phytotoxic than the individual compounds, concentration addition (CA) and independent action (IA) approaches were applied to estimate the predicted phytotoxicity values, followed by calculations of the MDR (model deviation factor) to determine the deviation from the predicted values. Binary mixtures of chloramphenicol with ketoprofen, diclofenac sodium, and oxytetracycline hydrochloride proved to be synergistic. The pH changes had the largest impact on the phytotoxicity of the diclofenac sodium and ketoprofen solutions, elevating their toxicity toward *S*. *bicolor*, and the co-presence of inorganic ions was shown to have an impact on ketoprofen, chloramphenicol, and oxytetracycline hydrochloride. Most of the interactions between sorgo plants and pharmaceuticals with added ions were antagonistic in nature, particularly those calculated using the IA model, with a few cases (one case for ketoprofen and chloramphenicol, two cases for oxytetracycline hydrochloride, and four cases for diclofenac sodium) of overestimation.

**Electronic supplementary material:**

The online version of this article (10.1007/s11356-018-2049-4) contains supplementary material, which is available to authorized users.

## Introduction

During wastewater treatment processes, drugs are often not fully degraded, for example, the elimination yield of diclofenac varies between 30 and 70% for conventional methods of wastewater treatment, such as in sewage residues containing pharmaceuticals entering into the environment (Lonappan et al. 2016). Additionally, a common practice is to apply sewage sludge (biosolids) as fertilizers in arid and semi-arid areas (Bartrons and Peñuelas 2017). Unfortunately, the safeness of biosolids is debatable due to their large content of, among other compounds, non-steroidal anti-inflammatory drugs (NSAIDs), anticonvulsants, and other pharmaceuticals and personal care products (PPCPs), which can be transferred directly to the ground during fertilization.

**Fig. 1:**
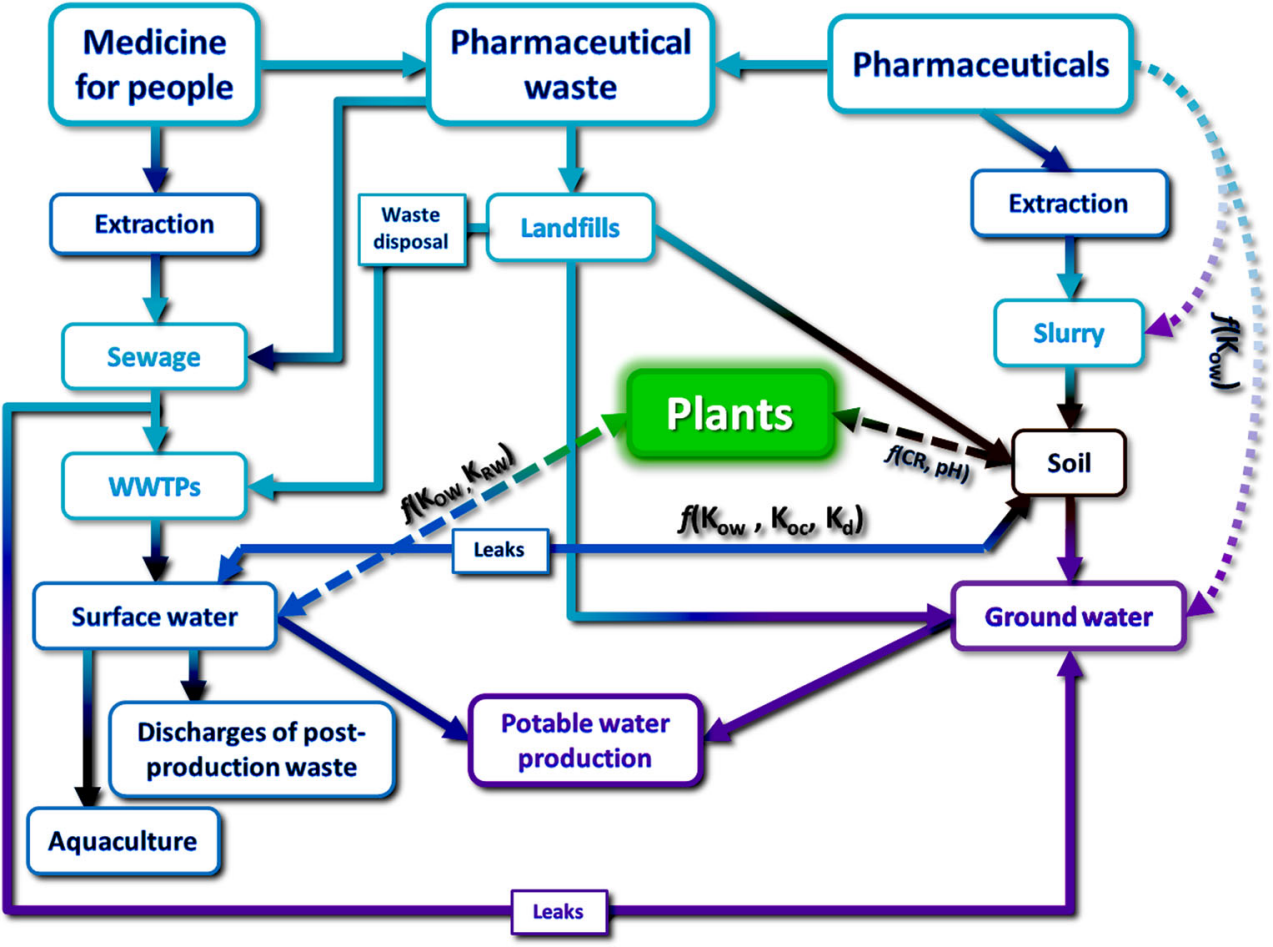
Schematic representation of pathways and sources of pharmaceuticals in the environment. K_ow_—octanol-water partition coefficient, K_oc_—organic carbon to water partition coefficient, K_RW_—root to water partition coefficient, K_d_—solid/liquid partition coefficient, CR—plant/soil concentration ratio (Halling-Sørensen et al. 1998; Wieczerzak et al. 2016b)

Pharmaceuticals can also reach the environment in animal manure (overloaded with veterinary antibiotics), by the direct application of agents to the ground or water (e.g., oxytetracycline is added to ponds during fish breeding) or as contaminated effluent coming from cemeteries or illegal landfills. Possible paths of penetration and sources of pollution in the environment are shown in Fig. 1 (Focazio et al. 2008).

Due to their low volatility, pharmaceuticals percolate mainly with water or through soil particles. After being introduced into the soil, drugs undergo sorption and biotic (microbial) or abiotic transformation processes. These processes may affect the concentration levels of pharmaceutical fractions available to plant roots. Due to the lack of access to light in deeper parts of the soil, processes such as photolysis do not play a role in the degradation of pharmaceutical residues. However, drugs in soil may undergo hydrolysis and oxidation processes. These processes have an impact on the formation of non-exchangeable or bound pharmaceutical residues, which are less bioavailable (Li et al. 2013).

The pharmaceutical fraction available to plants in soils may vary depending on the microbial composition present, for example, drugs in the form of second phase metabolites can be deconjugated as the result of microorganism actions, which increase the amount of the original drug (Miller et al. 2016). The exchangeable and dissolved parts of pharmaceuticals can reach ground water and be uptaken by plants (Semple et al. 2004).

Plants absorb drugs through roots mainly as a result of mass flow or via diffusion. Neutral pharmaceuticals can penetrate across the membranes of root cells, and ionizable compounds penetrate into plant tissues through a combination of diffusion processes and electrostatic interactions (Bartrons and Peñuelas 2017; Miller et al. 2016). The difference in the physicochemical properties of particular pharmaceuticals impact bioaccumulation processes in plants, especially acidity and alkalinity or lipophilicity. For example, some studies found that basic pharmaceuticals tend to translocate from the roots to the stalks and leaves more than neutral or acidic ones. Knowledge about metabolic and other processes that can occur in plants after exposure to drugs is limited, and not many studies have investigated this topic (Wu et al. 2015).

Pharmaceutical residues delivered from urban, agricultural, and industrial wastes can accumulate in soil and water at concentration levels ranging from nanograms per liter to micrograms per liter or kilograms per liter, at which they can be toxic to plants. In most countries, this group of pollutants is not monitored on a regular basis, although there are a number of international programs, such as the European Medicines Agency (EMEA/CHMP/SWP/4447/00, EMA/CVMP/ERA/418282/2005), concerning research on individual pharmaceuticals of human and animal origin (Wieczerzak et al. 2015; Kudłak et al. 2011; EMA 2006, 2011).

Many published studies address the ecotoxicity of individual pharmaceuticals on higher plants and algae; however, it should be acknowledged that drugs in the soil are mixtures with a total toxicity that may be different than the sum of the individual drug effects. Bearing in mind the above, efforts were undertaken to study and provide information on the toxicity levels of selected pharmaceuticals against *Sorghum bicolor* seeds, to determine possible interactions occurring between the compounds in the binary mixtures, and to investigate the influence of ions and pH changes on the toxicity of the selected drugs. Table 1 shows information on the amount of the selected pharmaceuticals in the environment and their physicochemical properties.

**Table 1:** Information about pharmaceuticals selected for research

Analyte name	Biological activity	M_w_ [g/mol]	LogP—octanol/water partition coefficient	logK_d_—solid/liquid partition coefficient, matrices	Occurrence in the environment (in soil)	Determined concentration levels of pharmaceuticals in plants
Diclofenac (sodium salt)	A non-steroidal anti-inflammatory drug (NSAID) with an analgesic, anti-inflammatory, and antipyretic activity. Acts primarily by selectively inhibiting cyclooxygenase (COX-1) (Knopp et al. 2007)	318.13	0.6–0.8 experimental at the pH ≥ 10 (Ingram et al. 2011)	1.26–2.18 and 0.39–1.56 thermophilic and mesophilic digested sludge (Carballa et al. 2008) 0.68–0.74 sediments from Dobroczyn basin, Poland (Styszko 2016)	4–92^a^ ng/g river sediments, Túria River, Spain (Carmona et al. 2014) 0.1–0.3 μg/g soil cores, Jerez de la Frontera and Guadalete alluvial aquifer, Spain (Corada-Fernández et al. 2015)	LOD—19 ng/g lettuce—dry weight (Calderón-Preciado et al. 2013) Average 17.8 ng/g eggplant—dry weigh (Riemenschneider et al. 2016)
Ketoprofen	A non-steroidal anti-inflammatory drug (NSAID) with anti-inflammatory, analgesic, and antipyretic activity. Drug acts by inhibiting both the constitutive (COX-1) and inducible (COX-2) types of cyclooxygenase (Thyss et al. 1986)	254.28	3.12–3.16—model simulation (Tixier et al. 2003)	0.66–1.07 foam-textured soil (Zhang et al. 2017) 0.11–0.30 sediments from Dobroczyn basin, Poland (Styszko 2016)	max. 97.35 ng/g natural, industrial, and agricultural soil, Cartagena-La Unión (Murcia), forest area of Monte de las Cenizas (Gutiérrez et al. 2016)	Data not available
Chloramphenicol	An antibiotic agent produced by *Streptomyces venezuelae.* The antibacterial action occurs by blocking the biosynthesis of protein in the ribosome (Kalkhambkar et al. 2013)	323.13	0.92–1.28—program predictions (KowWin^®^, ACD, ClogP^®^) (Machatha and Yalkowsky 2005)	0.39 agricultural soil, Hong Kong, China (Pan and Chu 2016	nd—2.31 ng/g sea sediments coast of Dalian, China (Na et al. 2013) 2.6–22.4 μg/kg crops, Pearl River Delta, southern China (Pan et al. 2014)	nd—10.1 ng/g spinach, leaf and root (Pan et al. 2014) 0.1–450 ng/g herbs, e.g., *Artemisia frigida* and *Thalictrum simplex*, collected in Mongolia, USA, and UK (Berendsen et al. 2010)
Oxytetracycline hydrochloride	An antibiotic agent that inhibits protein synthesis by blocking the attachment of charged aminoacyl-tRNA to the A site on the ribosome (Ciak and Hahn 1958; Jones and Morrison 1962)	496.89	1.12—experimental (ter Laak et al. 2006)	1.92 (1.87–1.96) (manure) 2.84 (2.6–3.0) sandy soil—sandy loam soil (ter Laak et al. 2006)	124–2683^b^ ng/g soil, Tianjin, China (Hu et al. 2010) Average 2.34^c^ ng/g river sediments, Liao River, China (Zhou et al. 2011) 14–0.52^b^ ng/g sediments, Yangtze Estuary, China (Shi et al. 2014)	35–330^b^ ng/g coriander 8.3–57^b^ ng/g radish 56–187^b^ ng/g rape (Hu et al. 2010) LOD—23.0 ng/g carrot, root (Pan et al. 2014).

*nd* not detected, *LOD* limit of detection

^a^Values detected for diclofenac

^b^Values detected for oxytetracycline

^c^Mean values

Studies on the effects of interactions occurring between drug mixtures and plants are rare; however, from the point of view of the delicate balance prevailing in ecosystems, the topic seems to be important; pharmaceuticals are present in the environment in mixtures with other pharmaceuticals and pollutants. Therefore, an additional distinctive feature of this study is research presenting the influence of certain environmental factors on the phytotoxicity of pharmaceuticals in higher plants.

The obtained results were mathematically treated to estimate the possible interactions in a way that is comparable to the results of other studies dealing with similar research. Environmental pollution with pharmaceuticals (and their residues) may lead to irreversible changes in ecosystems and affect the long-term quality of human lives, so it is important to conduct comprehensive research on and recognize the dangers of environmental pollution. To the best of our knowledge, this is one of very few studies dealing with the determination of the toxicity of binary drug mixtures against *S*. *bicolor* seeds.

## Materials and methods

### Chemicals, reagents, and instruments

#### Model substances selected for the study

Diclofenac (sodium salt) (CAS no. 153907-79-6), chloramphenicol (CAS no. 56-75-7), oxytetracycline h. (hydrochloride) (CAS no. 2058-46-0), fluoxetine h. (hydrochloride) (CAS no. 56296-78-7), estrone (CAS no. 53-16-7), ketoprofen (CAS no. 22071-15-4), progesterone (CAS no. 57-83-0), gemfibrozil (CAS no. 25812-30-0), and androstenedione (CAS no. 63-05-8) were purchased from Sigma-Aldrich (Germany) and were of analytical purity grade (> 99%). Diazepam (> 99% purity, CAS no. 439-14-5) was purchased from LGC Standards (UK). Inorganic ions in the form of respective salt (KCl (CAS no. 7447-40-7), NH_4_Cl (CAS no. 12125-02-9), NaF (CAS no. 7681-49-4), NaBr (CAS no. 7647-15-6), > 99% purity) were purchased from Avantor Performance Materials S.A. (Poland) while NaCl (CAS no. 7647-14-5, > 99% purity) was purchased from Sigma-Aldrich (Germany). pH values were adjusted with NaOH (CAS no. 1310-73-2, > 99% purity) or HCl (CAS no. 7647-01-0, > 99% purity) purchased from Avantor Performance Materials S.A. (Poland). *S*. *bicolor* seeds, black paper filters, and plastic containers were purchased from MicroBioTests Inc. (Belgium). Cotton wool used as subsoil was purchased from local drugstore (in order to maintain reproducibility the type and brand of wool was always the same). Pictures of germinated seeds were processed using freely available *ImageJ* software. pH was measured with Metrohm model 827 pH.

### Calculation of growth inhibition of *Sorghum bicolor* seeds

The phytotoxicity biotest principle relies on measuring the growth inhibition of young roots of seeds sprouting after 3 days of exposure to toxic substances or contaminated soil and comparing the results with seed growth on a control plate with reference soil and distilled water. The assay utilizes flat plastic containers consisting of two chambers; in the lower chamber, cotton wool is saturated with the test solution/control (distilled water). The experimental set is covered with a black paper filter, and ten seeds are placed (at equal distance separation) on its top edge. The plate is covered with a transparent lid, placed in a vertical position, and incubated at room temperature for 3 days. The incubation period is followed by image capture, and images are analyzed with free *ImageJ* software.

To determine the amount of cotton wool and test solution volumes required for the phytotoxicity research, a water holding capacity (WHC) test was performed. Seven grams of cotton wool was saturated with 50 cm^3^ of distilled water. After a few minutes of waiting, the water that did not soak into the cotton was removed and measured. It was determined that 18 cm^3^ of solution was sufficient to completely saturate the cotton wool. For all stages of the study, the procedure remained the same; only the concentrations of the pharmaceuticals, added ions, and solution pH varied. Growth inhibition was calculated according to the instructions of the Phytotoxkit test using Eq. 1 (Phytotoxkit 2004):

1 $$ EC=\frac{{\mathrm{L}}_{\mathrm{C}}-{\mathrm{L}}_{\mathrm{S}}\ }{{\mathrm{L}}_{\mathrm{C}}} $$

where

- EC stands for root growth inhibition [%]
- L_C_ is an average increase in root length of the control sample [mm]
- L_S_ is an average increase in root length of the sample studied [mm]

### Determination of the EC_50_ of selected pharmaceuticals, toxicity of pharmaceutical mixtures, and effect of pH changes and ion additions

**Fig. 2:**
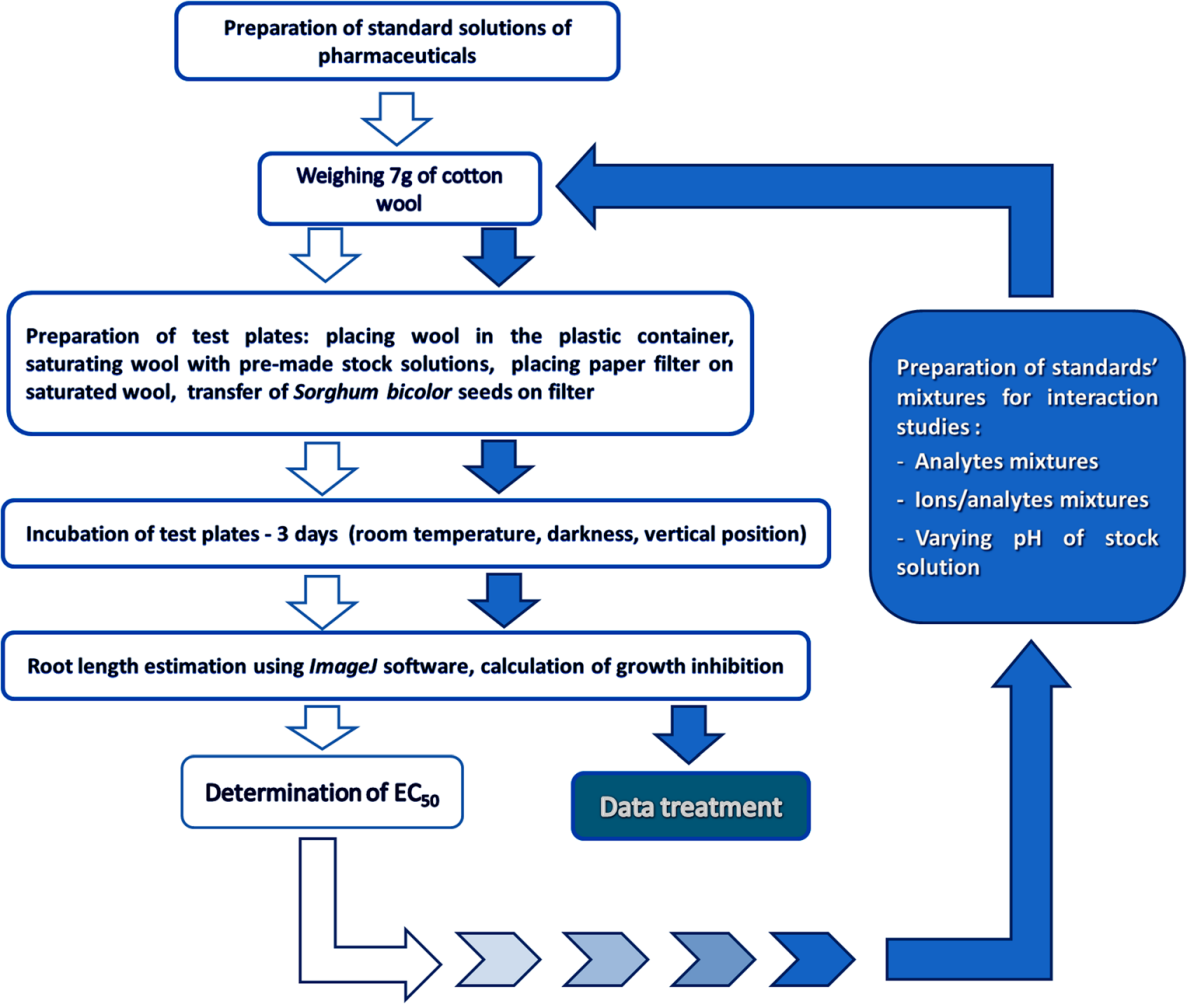
Schematic presentation of research methodology

To determine the effective concentration of the root growth inhibition (EC_50_) parameter, diluted solutions of the pharmaceuticals were added to the test plates, and each concentration was studied in triplicate, following the procedure described above, which is also shown in Fig. 2. After the 3-day incubation period, for some substances, no growth inhibition was observed, even at very high concentrations of the substances (above which they became insoluble in water). Therefore, it was impossible to determine the EC_50_ values of these substances; consequently, the toxicity studies had to be terminated at the concentrations given below:

- Progesterone—NOEC = 0.83 mg/L
- Estrone—NOEC = 189.10 mg/L
- Androstenedione—NOEC = 147.98 mg/L
- Fluoxetine h.—LOEC = 492.06 mg/L
- Gemfibrozil—LOEC = 0.15 mg/L
- Diazepam—LOEC = 0.099 mg/L

Having performed the calculation of root growth inhibition for each concentration studied, the results were used to form dose-response curves for each chemical studied (ketoprofen, oxytetracycline h., diclofenac sodium, and chloramphenicol). The results obtained were subjected to logarithm transformation, and the EC_50_ values, which are listed in Table 2, were calculated from the curve equation; additionally, the curves and equations from which the EC_50_ values were calculated can be found in the supplementary materials (EC_50_ calculations).

**Table 2:** Comparison of EC_50_ values for the concentration of pharmaceuticals tested

Analyte	EC_50_ [mg/L]	
Chloramphenicol	1337.754 ± 0.052	
Oxytetracycline hydrochloride	199.53 ± 0.75	
Diclofenac sodium	782.11 ± 0.54	
Ketoprofen	422.94 ± 0.83	
	Ions studied	
Ion	Ion lower concentration values [mg/L]	Ion upper concentration values [mg/L]
Na^+^	25.06	200.01
K^+^	4.69	30.10
NH_4_^+^	0.76	10.10
Cl^−^	24.82	303.83
F^−^	0.42	0.49
Br^−^	1.04	5.03

The next stage of the study was to determine whether the addition of ions at levels occurring in the environment would affect the toxicity of pharmaceuticals. Ion concentrations were selected according to research on water samples collected in water bodies receiving effluents from sewage treatment plants in Poland, as described in Kudłak et al. (2016) and listed in Table 2. Plates (prepared as previously described) were saturated with solutions of the chosen pharmaceuticals and ions such that the final concentrations were those given in Table 2. Plates with distilled water were used as the controls; additionally, to calculate the “expected” values used in the model deviation ratio (MDR) (described below), separate plates of pharmaceuticals and ions were used to determine the expected values (detailed data in supplementary materials). After the 3-day incubation period, root length was measured, and growth inhibition was calculated.

As reported by Kudłak et al. (2016), environmental water samples (collected from numerous locations in Poland) had pH values ranging from 4.93 to 8.15, with an average value of 7.15 (Kudłak et al. 2016). Therefore, in these studies, an attempt was also made to determine whether (and, if so, to what extent) the change in pH affects the toxicity of given pharmaceuticals. For this purpose, solutions of pharmaceuticals (ref. to Table 2) were subjected to pH adjustments with concentrated HCl or NaOH. The solutions were adjusted to seven different pH values ranging from 5.5 to 8.5 (at 0.5 steps), and the pH was measured with a pH meter. The cotton wool on the test plates was saturated with the pH-fixed pharmaceutical solutions as previously described. For the control, distilled water and pharmaceutical solutions without pH adjustments were used. After the 3-day incubation period, root length was measured, and growth inhibition was calculated.

As residues of pharmaceuticals in the environment do not occur separately but occur in mixtures with other substances, the last stage of the study examined the toxicity of two binary mixtures. Research was carried out for various ratios of the previously determined EC_50_ values for different pharmaceuticals, e.g., the first substance at 100% of its EC_50_ concentration was mixed with the second substance at 100% of its EC_50_ concentration, or the first substance at 50% of its EC_50_ concentration was mixed with the second substance at 150% of its EC_50_ concentration. Distilled water was used as the control, and individual pharmaceuticals at 50, 100, and 150% of their EC_50_ concentrations were also studied to confirm the test validity. All studies were performed in duplicate, except for the EC_50_ determination research, in which studies were performed in triplicates; ten *S*. *bicolor* seeds were used for each experiment. As already stated, after a 3-day incubation period, the root length was measured, and the growth inhibition was calculated.

### Model deviation ratio calculations

Many approaches for calculating the environmental risks resulting from exposure to pollutant mixtures can be found in scientific publications. Concentration addition (CA) and independent action (IA) approaches are among the most commonly used models. Each of these models is characterized by a different mode of action (MOA) of tested substances in the mixtures. The term MOA specifies the most important processes based on interactions with a specific receptor that are affected by changes in an organism and result in harmful or even lethal effects. The premise of CA modeling has a similar MOA as toxicants, as opposed to IA, where toxins act independently (Belden et al. 2007; Wieczerzak et al. 2016a). CA models are more frequently applied than IA models, although the latter are more accurate; however, in most cases, the differences in the CA and IA results are negligible.

In this study, the combined toxicological effect of a mixture of pharmaceuticals and the impact of environmental conditions on *S*. *bicolor* seeds were assessed with both the CA and IA models using Eqs. 2 and 3, respectively (Faust et al. 2000):

2 $$ {EC}_{X_{\mathrm{Mix}}}={\left({\sum}_{i=1}^n\frac{P_i}{EC_{x_i}}\right)}^{-1} $$

where

- *ECx*_mix_ is the *x*_mix_ effect cause by the total concentration of the mixture of studied chemicals (components) (expected value)
- *p*_*i*_ indicates the fraction of component *i* in the mixture, calculated on the basis of the concentration of *i* in the mixture
- *n* indicates the number of components in the mixture
- *ECx*_*i*_ indicates the *x*_*i*_ effect caused by component *i* at a given studied concentration in the mixture

3 $$ E\left({C}_{\mathrm{mix}}\right)=1-{\prod}_{i=1}^n\left(1-E\left({c}_1\right)\right) $$

where

- *EC*_mix_ is the overall effect expressed as a fraction of the maximal possible effect of a mixture of *i* chemical (expected value)
- *c*_*i*_ indicates the concentration of component *i* in the mixture
- *n* indicates the number of components in the mixture
- *E*(*ci*) indicates the effect of component *i*, applied separately

The application of the CA and IA models alone does not allow for the determination of possible interactions between chemicals (in this case, pharmaceuticals) in the mixture, and it does not define their character. The deviation from the predicted effect of the CA and IA models may be evidence for the occurrence of synergistic or antagonistic actions. To verify the difference between the predicted and observed effects, the MDR approach was applied, as defined by Eq. 4 (Wieczerzak et al. 2016a):

4 $$ MDR=\frac{\mathrm{Expected}}{\mathrm{Observed}} $$

where

- *Expected* is the effective toxicity (raw values of root growth inhibition calculated according to Eq. 1) of the mixture predicted by the CA or IA model
- *Observed* is the effective toxicity (raw values of root growth inhibition calculated according to eq. 1) for the mixture obtained during the toxicity studies

The mixtures with MDR > 2.0 exhibit a high probability of antagonism, while those with values below 0.5 show a synergistic character (Belden et al. 2007; Kienzler et al. 2016; Backhaus and Faust 2012; Faust et al. 2000). In the current research, it was arbitrarily assumed that an MDR falling within 0.50–0.71 and 1.40–2.00 justifies the conclusion of, respectively, the possible under- and overestimation of the presented models. It is worth noting that root growth inhibition values are used for MDR calculations, not the EC_50_ values themselves (in such case, the reverse values of MDRs would be obtained).

For studies on the impact of pH changes on pharmaceuticals solutions, the use of CA and IA modeling methods was impossible because these solutions cannot be treated as mixtures of chemicals. Therefore, in this case, a simple ratio was used (described by the Eq. 5) to assess the impact of the pH change:

5 $$ \mathrm{Effect}\ \mathrm{ratio}=\frac{\mathrm{Effect}\ \mathrm{observed}\ \mathrm{for}\ \mathrm{pharmaceutical}\ \mathrm{solutions}\ \mathrm{with}\ \mathrm{corrected}\ \mathrm{pH}}{\mathrm{Effect}\ \mathrm{observed}\ \mathrm{for}\ \mathrm{pharamceutical}\ \mathrm{solutions}\ \mathrm{with}\mathrm{out}\ \mathrm{pH}\ \mathrm{adjustment}\ } $$

Solutions of pharmaceuticals with a set pH for which the value of the ratio was lower than one were less toxic toward *S*. *bicolor*, and those with values above one were more toxic toward *S*. *bicolor.*

## Results and discussion

### Impact of inorganic ions on toxicity of selected compounds toward *Sorghum bicolor*

**Fig. 3:**
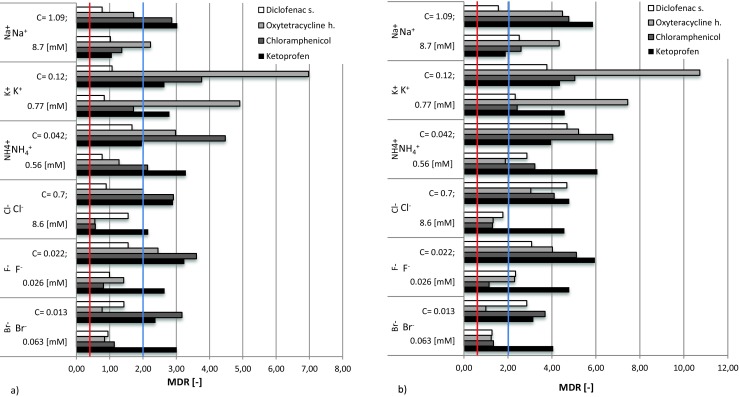
MDR values variations for **a** CA and **b** IA modeling for selected pharmaceuticals depending on co-presence of anions and cations at environmentally relevant levels (two concentration levels for each ion studied). All values above blue line (MDR = 2.0) confirm antagonistic character while all values below red line (MDR = 0.5) indicate synergic action

Generally, the results obtained for the studies on pharmaceuticals indicate that ions (both anions and cations) were antagonistic, with the strongest effect found for the mixture of oxytetracycline h. and potassium ions (refer to Fig. 3)a, b for details), while pictures of the seeding experiments are shown in the electronic supplementary Fig. 1. As expected, a shift in toxicity for the negatively ionizing chemicals from strongly antagonistic to either mildly antagonistic or overestimated was observed as the inorganic cation concentrations increased. The opposite phenomenon was generally observed for anions.

In the case of the impact of ions on chloramphenicol, overestimation and antagonistic behavior was confirmed in most cases. A similar situation was observed for oxytetracycline h., where bromides and chlorides were responsible for the underestimation and synergism; an antagonistic impact was observed in the case of most cations. Interestingly, in the case of diclofenac sodium, only two weak signals of underestimation were found, for a low concentration of sodium and a high bromide content. All other cases demonstrated situations of overestimation or antagonism. Such behavior was also true for the ketoprofen interactions with all ions studied (see Fig. 3).

### Impact of pH change on the toxicity of selected compounds toward *S*. *bicolor*

**Fig. 4:**
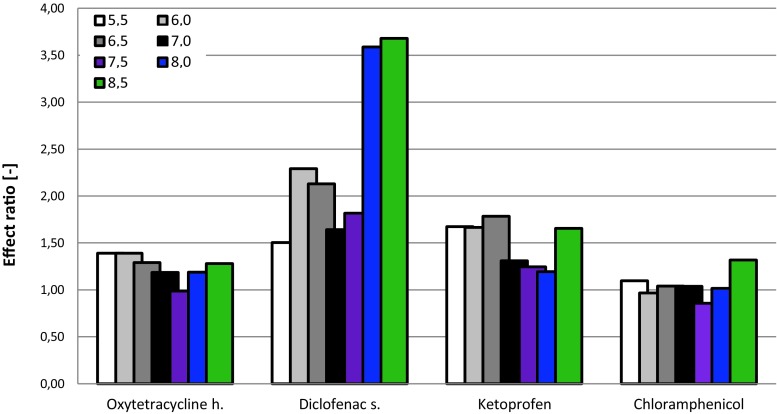
Impact of varying pH of pharmaceutical solutions on their phytotoxicity toward *S*. *bicolor*

Among the solutions tested, the change in pH had the greatest effect on the toxicity of diclofenac sodium; the toxicity of these solutions increased significantly at higher pH values (refer to Fig. 4). Zhao et al. (2017) observed that the adsorption of diclofenac to goethite depended on pH, and at lower pH values, diclofenac was adsorbed more quickly (equilibrium was achieved in a shorter time) and in greater amounts. This phenomenon could, in a sense, explain the lower toxicity of diclofenac sodium at lower pH values (see Additional Supplementary Fig. 2 for comparison); but due to the fact that another subsoil was used in mentioned toxicity study, it can not be said with certainty (Zhou et al. 2011; Zhao et al. 2017).

The pH change had a minor effect on the increase in the toxicity of ketoprofen solutions, particularly at pH values of 5.5, 6.0, 6.5, and 8.0. The toxicities of the oxytetracycline h. and chloramphenicol solutions were not affected by pH changes.

### Modeling of results of selected pharmaceutical binary mixture toxicity studies

**Fig. 5:**
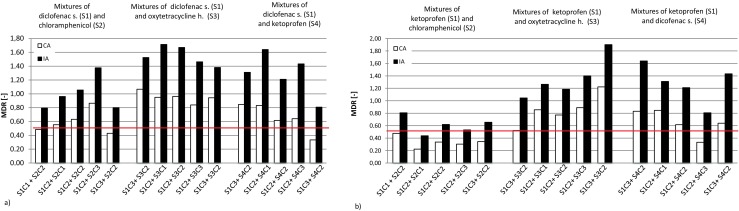
Predictions of MDR value variations for CA and IA models for mixture of **a** diclofenac sodium and **b** ketoprofen selected pharmaceuticals (chloramphenicol: C1 = 657.73 mg/L, C2 = 1337.75 mg/L, C3 = 1973.20 mg/L; oxytetracycline h.: C1 = 99.83 mg/L, C2 = 199.53 mg/L, C3 = 299.50 mg/L; diclofenac sodium: C1 = 391.15 mg/L, C2 = 782.11 mg/L, C3 = 1173.45 mg/L; ketoprofen: C1 = 211.49 mg/L, C2 = 422.94 mg/L, C3 = 634.46 mg/L). All values below red line (MDR = 0.5) indicate synergic action

**Fig. 6:**
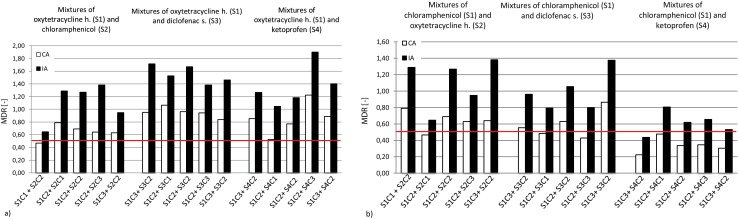
Predictions of MDR value variations for CA and IA models for mixture of **a** oxytetracycline h. and **b** chloramphenicol and selected pharmaceuticals (chloramphenicol: C1 = 657.73 mg/L, C2 = 1337.75 mg/L, C3 = 1973.20 mg/L; oxytetracycline h.: C1 = 99.83 mg/L, C2 = 199.53 mg/L, C3 = 299.50 mg/L; diclofenac sodium: C1 = 391.15 mg/L, C2 = 782.11 mg/L, C3 = 1173.45 mg/L; ketoprofen: C1 = 211.49 mg/L, C2 = 422.94 mg/L, C3 = 634.46 mg/L). All values below red line (MDR = 0.5) indicate synergic action

Chloramphenicol impacted all co-studied substances in a synergistic way; most cases of such behavior were observed for chloramphenicol and ketoprofen pairs by both the CA and IA models (see Fig. 5). The impact of oxytetracycline on varying concentrations of co-present pharmaceuticals in almost all cases was underestimated or overestimated, except for the case of a mixture with chloramphenicol, which is shown in Fig. 6.

For mixtures of drugs with diclofenac sodium, mostly independent actions of these chemicals were observed (under the conditions studied). Some tendency for synergism could be observed for the lowest concentrations of chloramphenicol and increasing concentrations of ketoprofen (see Fig. 5a, b). Ketoprofen had the most clearly observable synergistic impact on mixtures with chloramphenicol (refer to Fig. 6a, b). The situation was confirmed by both models used. No clear impact of this chemical could be shown in the case of mixtures with oxytetracycline (the independent action of these two chemicals is presumable), while a clear concentration impact trend was observable for studies with diclofenac (see example pictures in supplementary Fig. 3.).

## Conclusions

Pharmaceuticals and their residues are still considered to be newly emerging pollutants in the environment. Despite numerous studies on the qualitative and quantitative determination of pharmaceutical residues, still there is a strong need to combine instrumental and biological studies in environmental impact assessments. The results of the research presented herein enable the better understanding of the impacts these compounds have on living organisms under varying environmental conditions, which are complex and difficult to predict.

An attempt was made to assess the toxicity of drug interactions using a statistical approach to toxicity modeling presented with respect to the possible independent, antagonistic, or synergistic actions of drug and inorganic ion binary mixtures. One approach to the evaluation was the calculation of the MDR. Strong evidence proves the necessity of extrapolating the approach, as suggested for other organics considered to be pollutants, under environmentally relevant levels, as many of these pollutants have synergistic impacts on toxicity when present in complex mixtures; thus, it is almost impossible to predict toxicity with most of the models currently known and used. Furthermore, similar studies such as this one on *Sorghum bicolor* must be conducted for organisms from different trophic levels to fully understand the impact of pharmaceuticals on ecosystems.

The concentrations of drugs used in these studies, for which EC_50_ parameters were calculated, are significantly higher than the concentration levels measured in environmental samples. The use of such concentrations is justified to determine the EC_50_ values and to observe the moment of root growth inhibition. Nevertheless, it is possible that the interactions can occur at the cellular level and become toxic over longer exposure periods and at concentrations much lower than those studied.

## Electronic supplementary material

Fig. S1 (GIF 479 kb)

High Resolution Image (TIFF 22865 kb)

Fig. S2 (GIF 826 kb)

High Resolution Image (TIFF 32595 kb)

Fig. S3 (GIF 327 kb)

High Resolution Image (TIFF 13980 kb)

ESM 1 (DOCX 44 kb)
