# Supplementary figures and images for: Impact of selected drugs and their binary mixtures on the germination of Sorghum bicolor (sorgo) seeds
Source: Environ Sci Pollut Res Int. 2018 Apr 29;25(19):18717–27. doi: 10.1007/s11356-018-2049-4 (PMC6061511; doi:10.1007/s11356-018-2049-4)

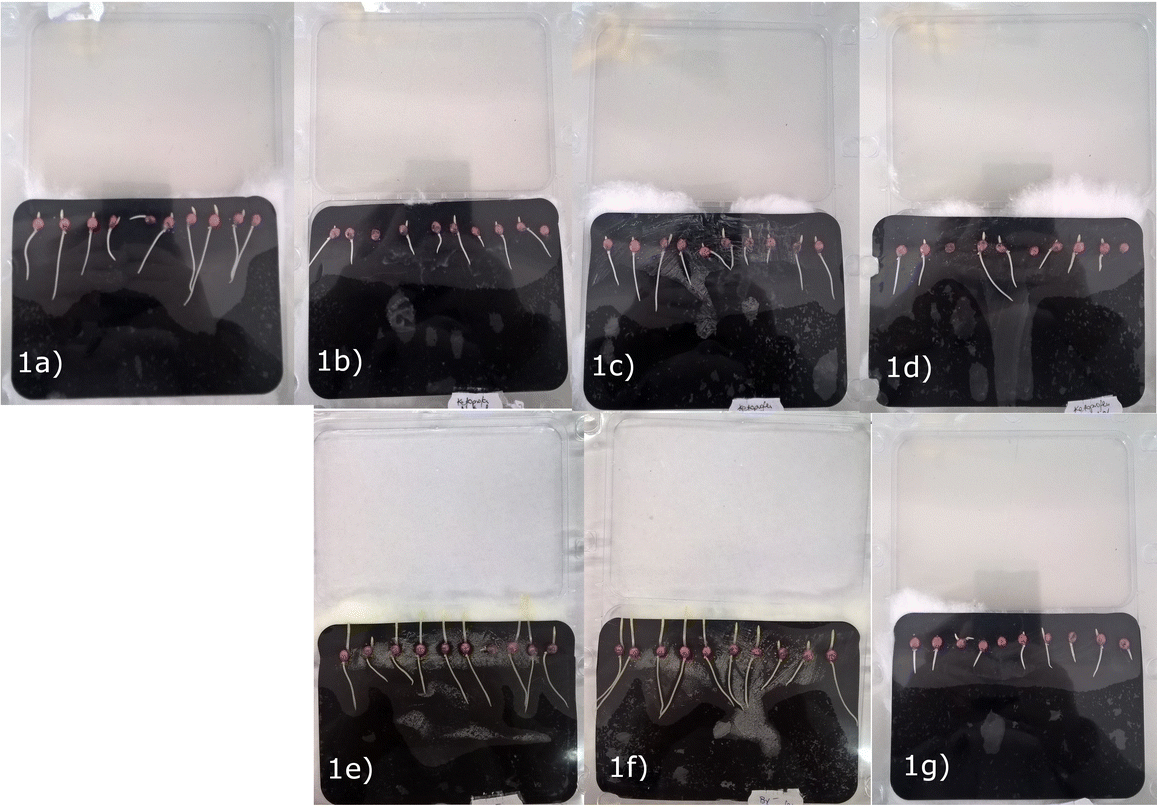

Supplement: Supplementary file 1 — (GIF 479 kb) [file 11356_2018_2049_Fig7_ESM.gif]

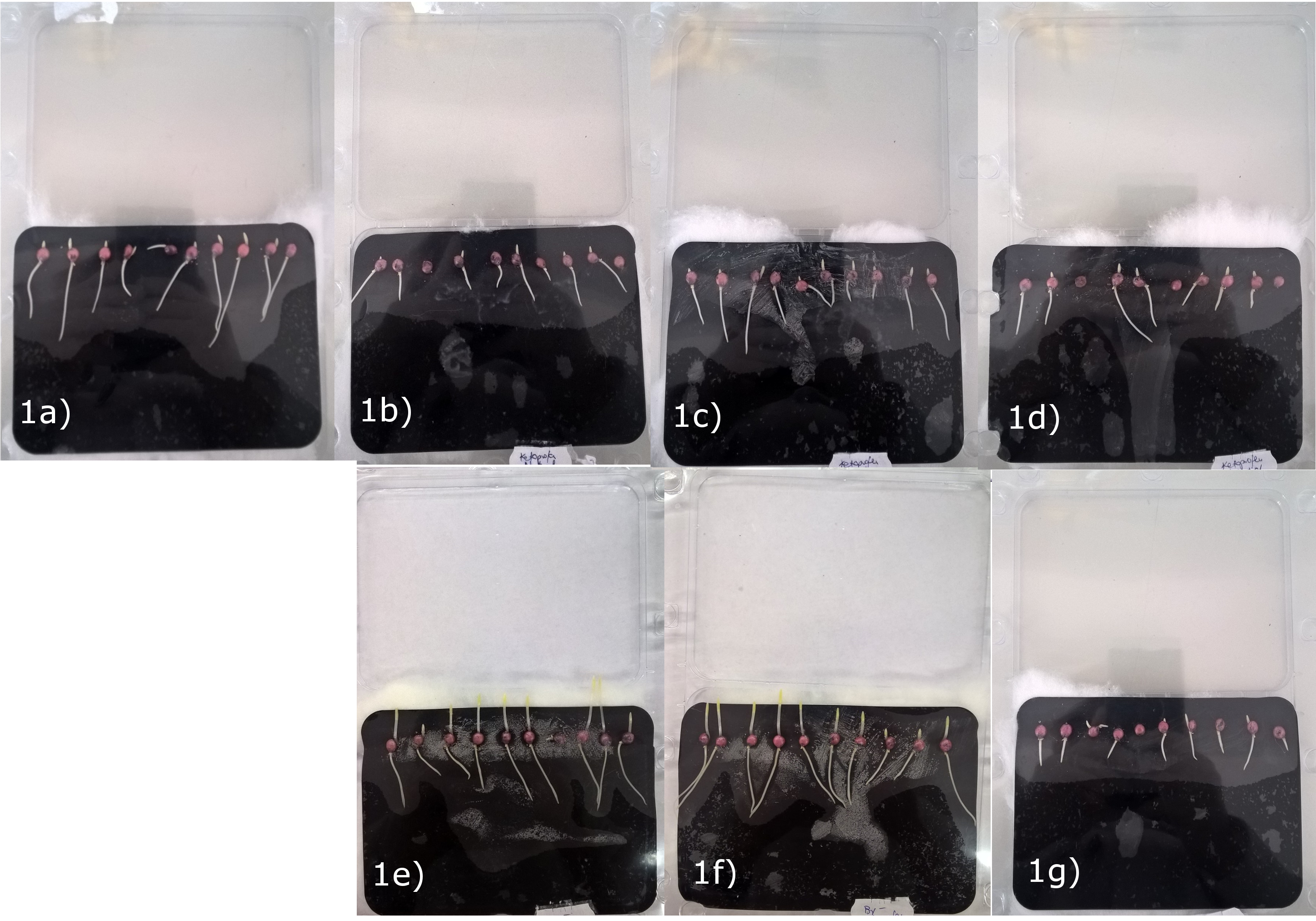

Supplement: Supplementary file 2 — High Resolution Image (TIFF 22865 kb) [file 11356_2018_2049_MOESM1_ESM.tiff]

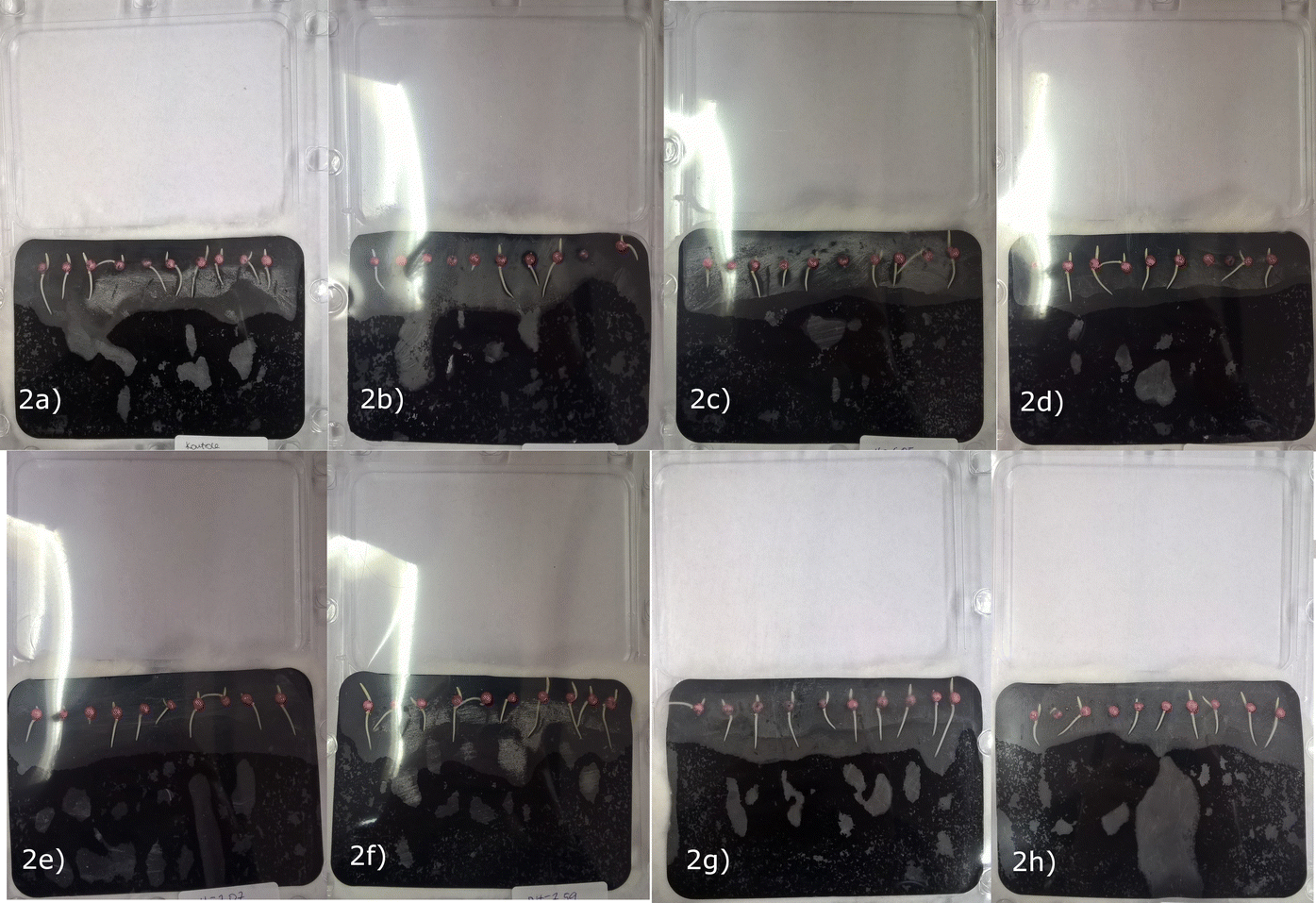

Supplement: Supplementary file 3 — (GIF 826 kb) [file 11356_2018_2049_Fig8_ESM.gif]

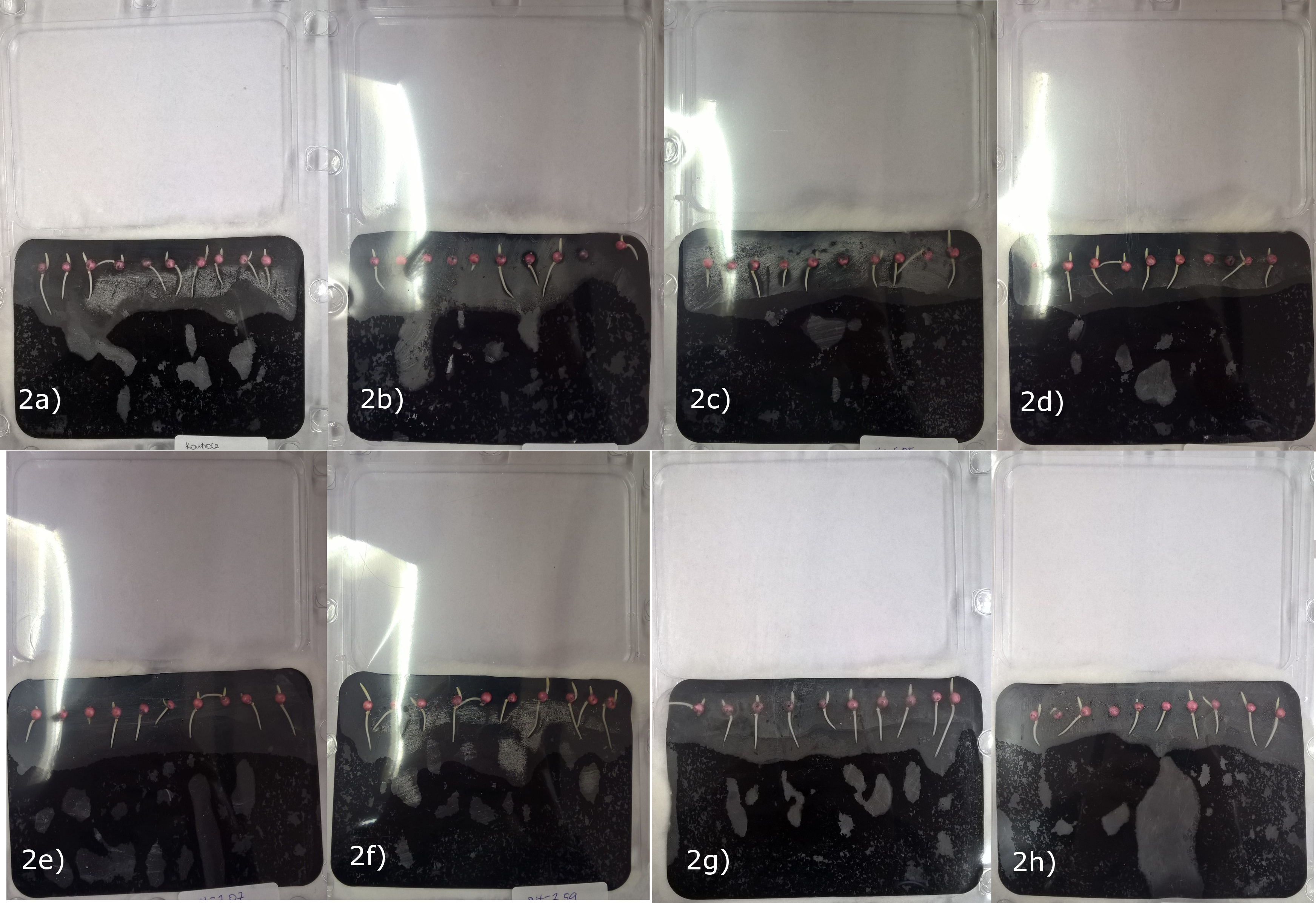

Supplement: Supplementary file 4 — High Resolution Image (TIFF 32595 kb) [file 11356_2018_2049_MOESM2_ESM.tiff]

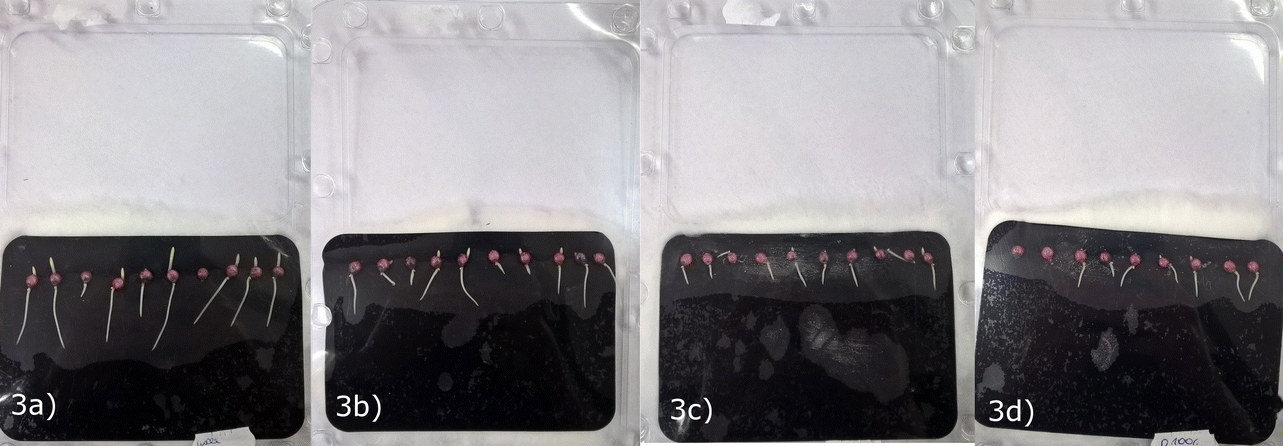

Supplement: Supplementary file 5 — (GIF 327 kb) [file 11356_2018_2049_Fig9_ESM.gif]

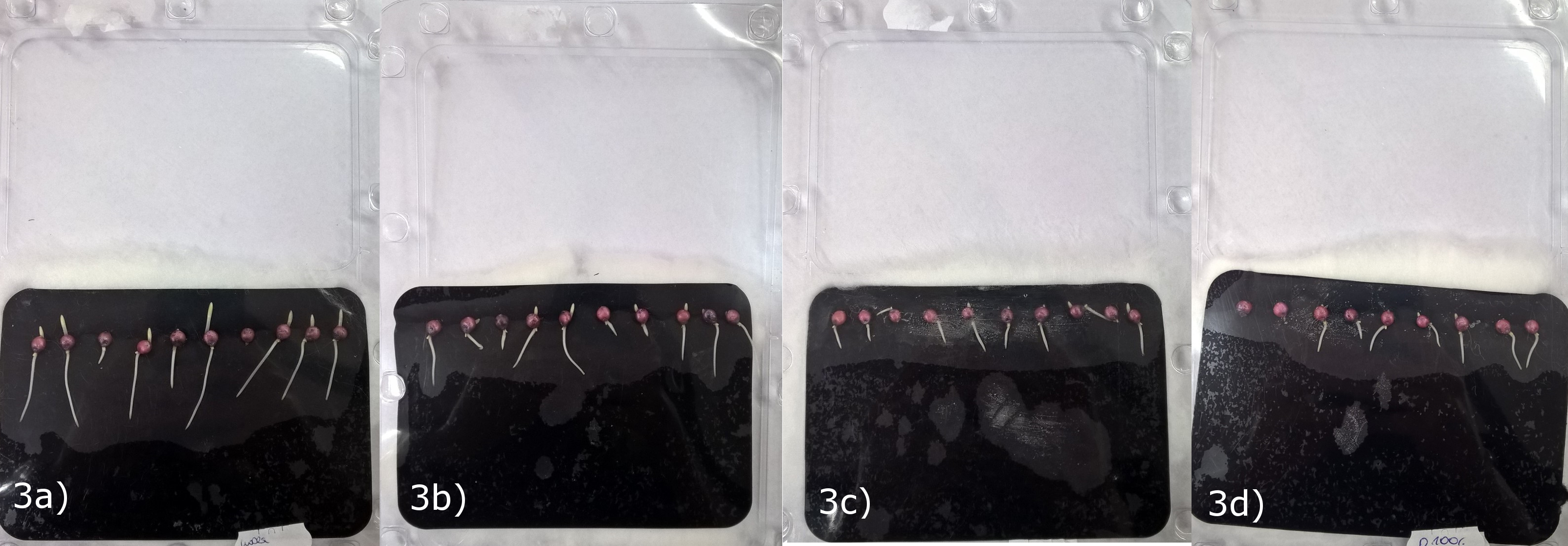

Supplement: Supplementary file 6 — High Resolution Image (TIFF 13980 kb) [file 11356_2018_2049_MOESM3_ESM.tiff]
