# Supplementary material for: Impact of selected drugs and their binary mixtures on the germination of Sorghum bicolor (sorgo) seeds
Source: Environ Sci Pollut Res Int. 2018 Apr 29;25(19):18717–27. doi: 10.1007/s11356-018-2049-4 (PMC6061511; doi:10.1007/s11356-018-2049-4)
Supplement: Supplementary file 7 — (DOCX 44 kb) [file 11356_2018_2049_MOESM4_ESM.docx]

*Electronic supplementary material for manuscript*:

Impact of selected drugs and their binary mixtures on the germination of *Sorghum bicolor* (sorgo) seeds

Monika WIECZERZAK^a^*, Błażej KUDŁAK^a^, Jacek NAMIEŚNIK^a^

^a^ Department of Analytical Chemistry, Faculty of Chemistry, Gdansk University of Technology, 11/12 Narutowicza Str., Gdańsk 80-233, Poland

*Corresponding author: monikawieczerzak66@gmail.com

2c. Methodology and data used in studies on EC_50_ determination for selected pharmaceuticals

1. Ketoprofen

To determine the EC_50_ parameter, diluted solutions of the ketoprofen were added to the test plates, each concentration was studied in triplicate, following the procedure described in the main text body and shown in Fig. 2. The results obtained were subjected to logarithm and response function was calculated as presented in Figure 2c.i. below. The EC_50_ value was calculated from curve equation, which was produced from logarithmized data listed in supplementary table 1. In case of ketoprofen the linear curve of negative logarithms of seed roots inhibition took form y = 0.3589x +0.1669. On this basis EC_50_ could be calculated as 10^(-log0.5+0.1669)/0.3589^= (422.94 ±0.83) mg/L.

| Supplementary Table 1. Data on response of *Sorghum bicolor* seeds root inhibition after subjection to given concentration levels of ketoprofen. | | |
| --- | --- | --- |
| Test no. | C_M_ [mg/mL] | Root growth inhibition [%]±SD |
| 1. | 0.00095 | 4.69±0.22 |
| 2. | 0.0095 | 12.106±0.045 |
| 3. | 0.048 | 24.77±0.11 |
| 4. | 0.095 | 43.174±0.032 |
| 5. | 0.19 | 42.187±0.021 |
| 6. | 0.29 | 45.564±0.037 |
| 7. | 0.38 | 47.624±0.025 |
| 8. | 0.48 | 56.740±0.021 |
| 9. | 0.76 | 54.222±0.020 |
| 10. | 0.95 | 64.525±0.077 |
| 11. | 1.05 | 60.203±0.067 |
| 12. | 1.53 | 71.7802±0.0073 |
| 13. | 1.62 | 80.395±0.014 |
| 14. | 2.58 | 91.1992±0.0072 |

Fig. 2c. i. Plot of negative logarithms of root growth inhibition in relation to ketoprofen concentration

1. Diclofenac (sodium salt)

To determine the EC_50_ parameter, diluted solutions of the diclofenac were added to the test plates, each concentration was studied in triplicate, following the procedure described in the main text body and shown in Fig. 2. The results obtained were subjected to logarithm and response function was calculated as presented in Figure 2c.i. below. The EC_50_ value was calculated from curve equation, which was produced from logarithmized data listed in supplementary table 2. In case of diclofenac the linear curve of negative logarithms of seed roots inhibition took form y=0.3001x+0.269. On this basis EC_50_ could be calculated as 10^(-log0.5+0.269)/0.3001^= (782.11±0.54) mg/L.

| Supplementary Table 2. Data on response of *Sorghum bicolor* seeds root inhibition after subjection to given concentration levels of diclofenac. | | |
| --- | --- | --- |
| Test no. | C_M_ [mg/mL] | Root growth inhibition [%]±SD |
| 1. | 0.0008837 | 9.522±0.028 |
| 2. | 0.008837 | 15.064±0.046 |
| 3. | 0.022092 | 12.220±0.058 |
| 4. | 0.04418 | 14.81±0.43 |
| 5. | 0.08837 | 25.293±0.013 |
| 6. | 0.17674 | 27.230±0.028 |
| 7. | 0.35348 | 32.892±0.092 |
| 8. | 0.53022 | 40.812±0.085 |
| 9. | 0.7070 | 38.314±0.043 |
| 10. | 0.8837 | 53.579±0.013 |
| 11. | 1.0604 | 59.041±0.038 |
| 12. | 1.2372 | 67.1746±0.0080 |
| 13. | 1.4139 | 79.472±0.026 |
| 14 | 1.5907 | 84.421±0.038 |

Fig. 2c. ii. Plot of negative logarithms of root growth inhibition in relation to diclofenac concentration

1. Chloramphenicol

To determine the EC_50_ parameter, diluted solutions of the chloramphenicol were added to the test plates, each concentration was studied in triplicate, following the procedure described in the main text body and shown in Fig. 2. The results obtained were subjected to logarithm and response function was calculated as presented in Figure 2c.i. below. The EC_50_ value was calculated from curve equation, which was produced from logarithmized data listed in supplementary table 3. In case of chloramphenicol the linear curve of negative logarithms of seed roots inhibition took form y=0.5061x-0.3614. On this basis EC_50_ could be calculated as 10^(-log0.5+0.3614)/0.5061^= (1337.754 ±0.052) mg/L.

| Supplementary Table 3. Data on response of *Sorghum bicolor* seeds root inhibition after subjection to given concentration levels of chloramphenicol. | | |
| --- | --- | --- |
| Test no. | C_M_ [mg/mL] | Root growth inhibition [%] |
| 1. | 0.0269 | 5.723±0.201 |
| 2. | 0.0539 | 13.013±0.058 |
| 3. | 0.1077 | 17.748±0.029 |
| 4. | 0.3231 | 18.904±0.114 |
| 5. | 0.4308 | 29.978±0.029 |
| 6. | 0.6463 | 31.022±0.037 |
| 7. | 0.8617 | 32.644±0.034 |
| 8. | 1.0771 | 40.399±0.031 |
| 9. | 1.2925 | 44.295±0.041 |
| 10. | 1.5079 | 53.747±0.050 |
| 11. | 1.7234 | 62.752±0.025 |
| 12. | 1.9388 | 87.5839±0.0071 |

Fig. 2c. iii. Plot of negative logarithms of root growth inhibition in relation to chloramphenicol concentration

1. Oxytetracycline

To determine the EC_50_ parameter, diluted solutions of the oxytetracycline were added to the test plates, each concentration was studied in triplicate, following the procedure described in the main text body and shown in Fig. 2. The results obtained were subjected to logarithm and response function was calculated as presented in Figure 2c.i. below. The EC_50_ value was calculated from curve equation, which was produced from logarithmized data listed in supplementary table 4. In case of oxytetracycline the linear curve of negative logarithms of seed roots inhibition took form y = 0.2789x – 0.1058. On this basis EC_50_ could be calculated as 10^(-log0.5+0.1058)/0.2786^= (199.53±0.75) mg/L.

| Supplementary Table 4. Data on response of *Sorghum bicolor* seeds root inhibition after subjection to given concentration levels of oxytetracycline. | | |
| --- | --- | --- |
| Test no. | C_M_ [mg/mL] | Root growth inhibition [%] |
| 1. | 0.0028 | 12.975±0.065 |
| 2. | 0.069 | 22.60±0.14 |
| 3. | 0.14 | 41.18±0.038 |
| 4. | 0.28 | 46.682±0.034 |
| 5. | 0.56 | 67.00±0.13 |
| 6. | 0.83 | 72.595±0.021 |
| 7. | 1.67 | 66.4616±0.0057 |
| 8. | 2.22 | 74.0679±0.0014 |
| 9. | 2.50 | 81.711±0.017 |
| 10. | 3.06 | 85.198±0.026 |

Fig. 2c. iv. Plot of negative logarithms of root growth inhibition in relation to oxytetracycline concentration

**Supplementary Fig. 1.**

Supplementary Fig. 1. Examples of pictures showing the toxicity of ketoprofen mixtures with ions against sorghum grains: 1a) water control, 1b) sodium ions, 1c) potassium ions, 1d) ammonium, 1e) chloride ions, 1f) fluoride ions, 1g) bromide ions.

**Supplementary Fig. 2.**

Supplementary Fig. 2. Examples of pictures showing the toxicity of diclofenac sodium solutions with adjusted pH: 2a) control solution (solution of diclofenac sodium without pH adjustment), 2b) pH = 5.5, 2c) pH = 6.0, 2d) pH = 6.5, 2e) pH = 7.0, 2f) pH = 7.5, 2g) pH = 8.0, 2h) pH = 8.5.

**Supplementary Fig. 3.**

Supplementary Fig. 3. Examples of pictures showing the toxicity of mixture of diclofenac sodium and oxyteracycline h.: 3a) distilled water control, 3b) mixture of diclofenac sodium (C3 = 1173.45 mg/L) and oxyteracyclne h. (C2 = 199.53 mg/L), 3c) control of diclofenac sodium (C3 = 1173.45 mg/L), 3d) control of oxyteteracycline h. (C2 = 199.53 mg/L).
